# An Improved Transformation System for *Phytophthora cinnamomi* Using Green Fluorescent Protein

**DOI:** 10.3389/fmicb.2021.682754

**Published:** 2021-07-05

**Authors:** Tingting Dai, Yue Xu, Xiao Yang, Binbin Jiao, Min Qiu, Junxin Xue, Felipe Arredondo, Brett M. Tyler

**Affiliations:** ^1^Co-Innovation Center for the Sustainable Forestry in Southern China, Nanjing Forestry University, Nanjing, China; ^2^Foreign Disease-Weed Science Research Unit, United States Department of Agriculture, Agricultural Research Service (ARS), Fort Detrick, MD, United States; ^3^ARS Research Participation Program, Oak Ridge Institute for Science and Education, Oak Ridge, TN, United States; ^4^Technical Center for Animal, Plant and Food Inspection and Quarantine of Shanghai Customs, Shanghai, China; ^5^Nanjing Agricultural University, Nanjing, China; ^6^Department of Botany and Plant Pathology, Center for Genome Research and Biocomputing, Oregon State University, Corvallis, OR, United States; ^7^Department of Botany and Plant Pathology, Oregon State University, Corvallis, OR, United States

**Keywords:** oomycete, GFP, *Phytophthora* root rot, pathogenicity, filamentous plant pathogen

## Abstract

*Phytophthora cinnamomi* is a destructive pathogen causing root rot and dieback diseases on hundreds of economically and ecologically important plant species. Effective transformation systems enable modifications of candidate genes to understand the pathogenesis of *P. cinnamomi*. A previous study reported a polyethylene glycol and calcium dichloride (PEG/CaCl_2_)-mediated protoplast transformation method of *P. cinnamomi*. However, the virulence of the transformants was compromised. In this study, we selected ATCC 15400 as a suitable wild-type isolate for PEG/CaCl_2_ transformation using the green fluorescent protein after screening 11 *P. cinnamomi* isolates. Three transformants, namely, PcGFP-1, PcGFP-3, and PcGFP-5, consistently displayed a green fluorescence in their hyphae, chlamydospores, and sporangia. The randomly selected transformant PcGFP-1 was as virulent as the wild-type isolate in causing hypocotyl lesions on lupines. Fluorescent hyphae and haustoria were observed intracellularly and intercellularly in lupine tissues inoculated with PcGFP-1 zoospores. The potential application of this improved transformation system for functional genomics studies of *P. cinnamomi* is discussed.

## Introduction

The genus *Phytophthora* contains over 100 plant pathogenic species that severely threaten agricultural production and natural ecosystems (Erwin and Ribeiro, 1996; Lamour et al., 2007; Kamoun et al., 2015), such as *Phytophthora infestans* causing late blight of potato (Fry and Goodwin, 1997), *Phytophthora sojae* causing root and stem rot of soybean (Schmitthenner, 1985), *Phytophthora ramorum* causing sudden oak death (Rizzo et al., 2002), *Phytophthora capsici* causing *Phytophthora* blight of many vegetable crops (Hausbeck and Lamour, 2004), and *Phytophthora cinnamomi*. *P. cinnamomi*, a devastating plant pathogen causing *Phytophthora* root rot and dieback diseases, has a global distribution and a large host range (Cahill et al., 2008; Jung et al., 2013; Hardham and Blackman, 2018). The diseases caused by *P. cinnamomi* can lead to not only enormous losses in agriculture and forestry but also a reduction in biodiversity (Hardham and Blackman, 2018).

Understanding the pathogenesis of *P. cinnamomi* is the key to the successful management of Phytophthora root rot and dieback. The identification of pathogenesis-related (PR) genes of *P. cinnamomi* has been rapidly advanced by the improvement of genome sequencing and bioinformatics technologies. Decoded by genome and transcriptome analyses, *P. cinnamomi* has at least 1,400 PR genes including approximately 32 putative elicitin genes, 29 putative Crinkler effector genes, and 266 RxLR effector candidate genes that are characterized by an Arg-Xaa-Leu-Arg (RxLR) motif (Meyer et al., 2016; Reitmann et al., 2017; Hardham and Blackman, 2018; Dai et al., 2020). Although a repository of PR genes of *P. cinnamomi* has been identified, the functions of the vast majority of these genes have not been determined (Hardham and Blackman, 2018; Dai et al., 2020). It will be crucial to determine the role of their encoded proteins during *in planta* infection. The central approach to this endeavor is applying transformation technologies to obtain the mutants of the studied PR genes.

Effective transformation systems and genome engineering strategies are required for functional analyses of PR genes of *Phytophthora* species. So far, transformation systems have been established for several *Phytophthora* species to study gene functions. For example, a polyethylene glycol and calcium dichloride (PEG/CaCl_2_)-mediated protoplast transformation method has been employed for *P. infestans* (Judelson et al., 1991), *Phytophthora palmivora* (van West et al., 1999), *P. ramorum* (Riedel et al., 2009), *P. capsici* (Dunn et al., 2013), *Phytophthora nicotianae* (Bottin et al., 1999; Meng et al., 2015), and *Phytophthora litchii* (Wang et al., 2019). *P. capsici*, *Phytophthora citricola*, *P. cinnamomi*, and *Phytophthora citrophthora* were transformed by microprojectile bombardment (Bailey et al., 1993). An electro-transformation system has been developed for *P. capsici* (Huitema et al., 2011), *P. infestans* (Latijnhouwers and Govers, 2003; Dong et al., 2015), and *P. palmivora* (Tyler, 2007). An *Agrobacterium*-mediated transformation method has been used for *P. infestans* (Vijn and Govers, 2003), *P. palmivora* (Wu et al., 2016), and *P. sojae* (Tyler, 2007). Furthermore, the genome editing technology CRISPR/Cas9 has been utilized for transforming proteins in *P. sojae* (Fang and Tyler, 2016), *P. palmivora* (Gumtow et al., 2018), and *P. capsici* (Wang et al., 2019). Although the PEG/CaCl_2_-mediated method was also used for the transformation of *P. cinnamomi* in a prior study, the vegetative growth of the transformants was reduced and the virulence on lupine roots was significantly lower than those of the non-transformed parent isolates (McCarren et al., 2005). Therefore, the transformed isolates were used for studying the growth and survival of *P. cinnamomi* in plants and soil under non-sterile conditions rather than for studying pathogenesis (McCarren et al., 2005). Horta et al. (2008) also indicated that *P. cinnamomi* was difficult to transform. In an effort to obtain lipofectin-mediated co-transformation of *P. cinnamomi* protoplasts with pHAMT35H and pHAMT35-fatss plasmids, only a single transformant carrying both plasmids was obtained, and it had lost approximately 50% virulence on *Quercus suber* roots (Horta et al., 2008).

The goal of the present study was to establish an improved PEG/CaCl_2_-mediated transformation system for *P. cinnamomi*. We aimed at generating transformants that were unchanged in vegetative development and virulence and thus could be used for downstream functional analyses of *P. cinnamomi* PR genes.

## Materials and Methods

### Selection and Virulence of *P. cinnamomi* Isolates

A total of 11 *P. cinnamomi* isolates from different regions and substrates (Table 1) were used for screening virulence. They were routinely cultured in 10% clarified V8 juice agar (cV8A) under a 12-h light/12-h dark cycle at room temperature (25°C) during this study.

**TABLE 1:** Information of *Phytophthora cinnamomi* isolates used for screening virulence.

**Isolate**	**Other isolate numbers**	**Host/Substrate**	**Location**	**Year**
ATCC 15400	ATCC MYA-4057	*Camellia japonica*	SC, United States	n.a.
1195		*Pieris* sp.	OR, United States	2015
7574		*Castanopsis* sp.	Taiwan	2015
CBS 144.22	ATCC 46671; IMI 22938	*Cinnamomum burmannii*	Indonesia	1922
ATCC 15401	ATCC MYA-4058	*Persea americana*	Puerto Rico	1960
Pc-1		*Rhododendron pulchrum*	Jiangsu, China	2013
7308		*Castanopsis* sp.	Taiwan	2015
7650		*Castanopsis* sp.	Taiwan	2015
3038		*Pieris* sp.	OR, United States	2015
1791		soil	OR, United States	2015
3764		*Pieris* sp.	OR, United States	2015

The virulence of these isolates was tested on detached *rhododendron* (*Rhododendron pulchrum*) leaves, apple (*Malus domestica* Borkh. cv. Red Fuji) fruits, and hypocotyls of 8-days-old potted lupine seedlings (*Lupinus angustifolius*). For rhododendron, six asymptomatic leaves were symmetrically wounded at both sides of the abaxial surface using a sterile inoculation needle. A 3-days-old cV8A plug (5 × 5 mm^2^) colonized by a *P. cinnamomi* isolate was placed on each wound of three leaves. Sterile agar plugs were placed on the wounds of three non-inoculated control leaves. After inoculation, all leaves were placed on a wet filter paper in a closed container (a moist chamber) and incubated at 25°C. Diameters of lesions developed surrounding the wounds were measured after 3 days.

For apples, two healthy fruits were stab wounded at the equator (approximately 5 mm deep). A 3-days-old *P. cinnamomi*-colonized agar plug (5 × 5 mm^2^) was inserted into the wound, while a sterile plug was used for a non-inoculated control fruit. Both inoculated and non-inoculated apple fruits were incubated at 25°C for 3 days, then lesion diameters surrounding the wounds were measured.

For lupines, 8-day-old seedlings were removed from pots and used for inoculation. Hypocotyls and roots were gently wounded using sterile pipette tips. Zoospores of each *P. cinnamomi* isolate were produced by cultivating mycelia in 10% clarified V8 juice liquid medium at 25°C in the dark for at least 3 days. Sporangia were induced by washing mycelial mats with sterile distilled water (dH_2_O) and incubating at 25°C in the dark. Zoospores were released by placing the mycelial mats in 10 ml of chilled sterile dH_2_O (4°C) for 30 min. Ten microliters of zoospore suspension (10^4^/ml) was pipetted onto a wound on each of the three inoculated hypocotyls. Sterile dH_2_O was used for a control hypocotyl. All seedlings were incubated in a moist chamber at 25°C for 3 days until lesion lengths along the hypocotyl and roots were measured. Additionally, to observe the expansion of *P. cinnamomi* hyphae in lupine tissues, three hypocotyls and roots were inoculated in the same manner as above using the isolate of the highest virulence. Samples were taken 6, 12, and 24 h after inoculation. The epidermal cells of the infected lupine were removed. Staining with a 0.05% Trypan Blue solution was followed by decolorization with chloral hydrate and observation under an optical microscope. The assays were repeated at triplicate times.

The above assays were repeated at triplicate times for each plant species and *P. cinnamomi* isolate combination. The average values of measurements across replications and repeated trials were calculated and analyzed for significance using Origin 2020 (OriginLab Corporation, Northampton, MA, United States).

### Construction of Plasmids

The oomycete expression vector pTOR (Blanco and Judelson, 2005; Wu et al., 2016) was used for the PEG/CaCl_2_-mediated transformation of *P. cinnamomi* (Supplementary Figure 1). Briefly, the bar gene expression cassette was removed using restriction enzymes *Kpn*I and *Sec*I and replaced with the fragment (1,011–4,018 nt) of the vector pTOR (GenBank Accession No. EU257520), which contains two gene expression cassettes. The first cassette contains the *Bremia lactucae* Ham34 promoter (1,011–1,575 nt), a multiple cloning site (1,576–1,688 nt) for cloning genes to be transformed, and a Ham34 terminator (1,689–2,209 nt). The second cassette contains the *B. lactucae* Hsp70 promoter (2,217–2,800 nt), the selective marker gene neomycin phosphotransferase *NPTII* (2,816–3,610 nt) used to select transformants resistant to aminoglycoside geneticin sulfate G418, and Hsp70 terminator (3,797–4,016 nt).

Primers pHam34-F-*Sac*I (5′-gcggagctcTCTGATGGACAAA GGGTCGCCT-3′) and THsp70-R-*Kpn*I (5′-gcgggtaccAAGCA CAATAGGCCCAGACTC-3′) were used to amplify this fragment using pTOR as template (Wu et al., 2016). The plasmid pTOR-GFP was generated by cloning the DNA fragment corresponding to the green fluorescent protein (GFP)-encoding sequence into the *Eco*RI and *Spe*I sites of pTOR. Primers GFP-F-*Eco*RI (5′-gcggaattcATGGTGAGCAAGGGCGAG-3′) and GFP-R-*Spe*I (5′-gcgactagtTTACTTGTACAGCTCGTCCATGC-3′) were used to amplify the GFP fragment (Wu et al., 2016).

### Preparation of Protoplast for Transformation

The PEG-CaCl_2_-mediated protoplast method described previously (Judelson et al., 1991) was used to transform *P. cinnamomi*, but with a few modifications. A lysing enzyme from *Trichoderma harzianum* (Product No. L1412, Sigma, St. Louis, MO, United States) and a CELLULYSIN^®^ cellulase from *T. viride* (Catalog No. 219466, Calbiochem, San Diego, CA, United States) were two important components of an enzyme solution for generating protoplasts for transformation. The solution also contained 0.4 M mannitol, 20 mM KCl, 20 mM MES buffer (pH 5.7), and 10 mM CaCl_2_. To determine the optimum concentrations of the lyase and the cellulase that could maximize protoplast yield at room temperature (25°C) with an incubation period of 50 min, a range of concentrations (2.5, 5, 10, 15, and 20 mg/ml) was tested for each enzyme, when the other enzyme was absent. The number of protoplasts with diameters of 15 ± 2.7 μm was calculated with a hemocytometer. The test for each enzyme was repeated three times.

The isolate selected from the above virulence screening was designated as the suitable wild type (WT) for the following transformation. It was cultured in 250-ml Erlenmeyer flasks containing 50 ml of nutrient pea broth at 25°C for 2.5 days (McLeod et al., 2008). Mycelial mats were harvested using autoclaved Miracloth (CalBiochem, La Jolla, CA, United States), rinsed in 40 ml of sterile deionized water and then 40 ml of 0.8 M mannitol. They were transferred into a Petri dish (25 mm deep) and immersed in 0.8 M mannitol at 25°C for 10 min for plasmolysis. Thereafter, plasmolyzed mycelia were transferred to 20 ml of the enzyme solution containing the optimum concentrations of lyase and cellulase and incubated at room temperature for 50 min with gentle shaking (55–60 rpm). The digestion products were filtered through a 70-μm Falcon nylon mesh cell strainer (BD Biosciences, San Jose, CA, United States) to remove mycelial debris. The flow-through was collected in 50-ml Falcon tubes and centrifugated at room temperature and 1,500×*g* for 4 min to pellet the protoplasts. After being washed with 30 ml of W5 solution (5 mM KCl, 125 mM CaCl_2_, 154 mM NaCl, and 173 mM glucose), the protoplasts were re-suspended in 7 ml of W5 solution and placed on ice for 30 min before being collected by centrifugation at 1,500×*g* for 5 min in 50-ml centrifuge tubes and re-suspended at 10^6^–10^7^/ml in MMg solution (0.4 M mannitol, 15 mM MgCl_2_, and 4 mM MES, pH 5.7).

The transformation was performed using 1 ml of protoplast suspension with 40–50 μg of DNA for single plasmid transformations or 20–30 μg of DNA for each plasmid for co-transformations. The protoplast-plasmid mixtures were incubated on ice for 30 min, before 1.74 ml of fresh PEG solution (40% PEG 4000 m/v, 0.2 M mannitol, and 0.1 M CaCl_2_) was gradually added. The tubes were then shaken gently and incubated on ice for another 20 min before the protoplasts were mixed with 20 ml of clarified pea broth and dark-incubated at 25°C for 20 h to allow regeneration.

### Screening Transformants

The regenerated protoplasts were collected by centrifugation at 1,500×*g* for 5 min and re-suspended in 35 ml of molten (45°C) pea broth agar (1.5% agar, 0.5 M mannitol) amended with Geneticin (G418) selective antibiotic (different concentration from 10 to 90 μg/ml), as *NPT II* was used as the selection marker. The medium containing the regenerated protoplasts was then divided into three Petri dishes and incubated at 25°C in the dark for 3 days to complete the first round of screening. Each plate was then covered with molten (45°C) cV8A medium (1.5% agar) amended with a suitable concentration of G418 and incubated under the same conditions for another 3 days. Colonies that appeared after this second round of screening were transferred to fresh cV8A plates amended with G418 for a third round of screening. To prevent bacterial contamination, 70 μg/ml of ampicillin was included in the medium used in each round of screening.

### Detection of GFP Transformants

The transformants grown on the selective agar media were cultured in a 70 μg/ml G418 selective liquid medium for 7 days at 25°C in the dark. PCR detections of the *GFP* gene in the G418-resistant transformants were performed using EasyTaq DNA Polymerase and 25-ml reaction mixtures containing 12.5 ml of 2 × Master Mix (Tsingke, Beijing, China), 0.5 ml of forward primer (10 mM), 0.5 ml of reverse primer (10 mM), and 200 ng of genomic DNA and cDNA. The primers were pTORG418-ORF-F (5′-CTGAATGAACTGCAGGACGA-3′) and pTORG418-ORF-R (5′-AATATCACGGGTAGCCAACG-3′). PCR reactions were conducted using a Bio-gener GT9612 thermocycler (Bio-gener, Hanzhou, China) and the following program: initial denaturing at 94°C for 4 min, followed by 34 cycles of denaturing at 94°C for 30 s, annealing at 58°C depending on the primers for 30 s, and extension at 72°C for 1 min, with a final extension at 72°C for 10 min. PCR products were visualized by 1% agarose gel electrophoresis. Each set of reactions included DNA samples of the WT and the empty vector (EV; pTOR) as the negative controls and the pTOR-GFP plasmid DNA as a positive control.

Green fluorescent protein expression was verified in different vegetative structures of the transformants including hyphae, chlamydospores, sporangia, and unreleased zoospores within cleaved sporangia using an Axio Imager A2m microscope (Carl Zeiss Microscopy, LLC, White Plains, NY, United States).

### *In vitro* Growth and *in planta* Virulence of the Transformants

To determine the growth rate, the selected transformants and the WT were cultured in 10% cV8A in 60-mm Petri dishes and incubated at 25°C in the dark for 3 days. The colony morphology was photographed. The radial growth of the colonies was measured. Four replication plates were used for each isolate. This experiment was repeated twice. All isolates were cultured and preserved in 10% V8 juice solid medium containing screening agent G418 (final concentration of G418 was 70 μg/ml) at 4°C temperature.

To determine the virulence, the transformants and the WT were used to inoculate detached rhododendron leaves, apple fruits, lupine seedlings, and roots in the same manner as described above. The lesions were measured on 2, 4, and 6 days after inoculation. The experiment was repeated three times. One-way analysis of variance (ANOVA) was carried out in Origin 2020 (OriginLab Corporation, Northampton, MA, United States) to compare the sizes of lesions induced by the transformant and the WT.

Microscopic analysis was also conducted to observe the GFP-labeled transformants in lupine tissues. Lupine hypocotyls were inoculated using the same method described above, except that 2-week-old lupines were used and 2 μl of zoospore suspension was pipetted onto each hypocotyl. The necrotic tissues surrounding the inoculation point was examined for GFP expression under the Axio Imager A2m microscope at 6, 12, and 24 h after inoculation.

## Results

### Isolate Selection for Transformation

**FIGURE 1:**
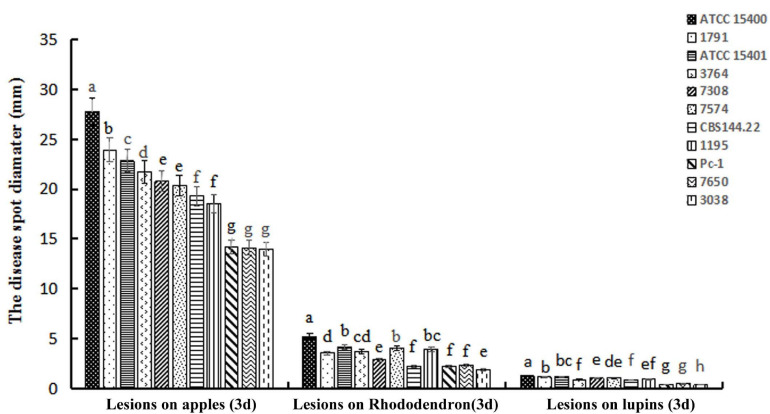
Average lesion sizes caused by 11 *Phytophthora cinnamomi* isolates on three plant species. Lesion diameters on apple fruits and rhododendron leaves and lesion lengths on lupine hypocotyls were measured 3 days after inoculation. Different letters above error bars for each host indicate a significant difference (*p* < 0.01) among isolates. Isolates from left to right for each host: ATCC 15400, 1791, ATCC 15401, 3764, 7308, 7574, CBS 144.22, 1195, Pc-1, 7650, and 3038.

**FIGURE 2:**
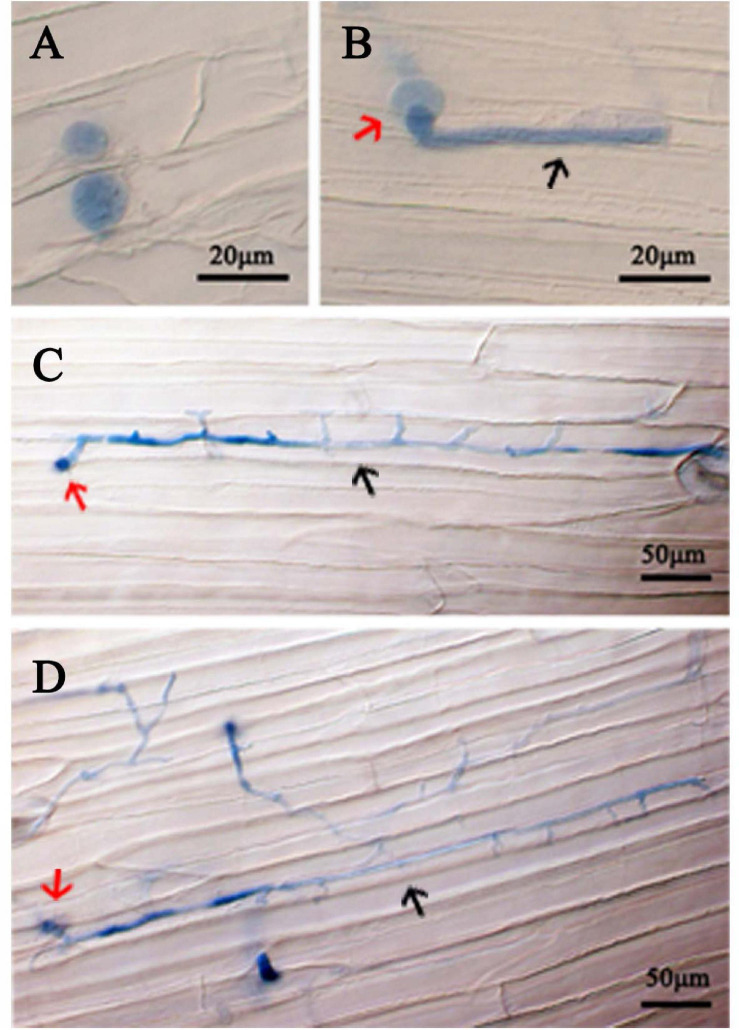
Colonization of *P. cinnamomi* isolate ATCC 15400 in lupine hypocotyl tissue. **(A)** Zoospores infecting hypocotyl tissue formed cysts 6 h after inoculation. **(B)** A cyst (red arrow) forming a short germ tube (black arrow) with an appressorium 6 h after inoculation. **(C)** A cyst (red arrow) forming an expanded germ tube (black arrow) 12 h after inoculation. **(D)** A cyst (red arrow) forming inter- and intra-cellular hyphae (black arrow) 24 h after inoculation. Cells of *P. cinnamomi* were stained using 0.05% Trypan Blue.

The virulence of 11 *P. cinnamomi* isolates was tested on three hosts. Lesion sizes measured in three replicate experiments, each with two to three inoculated plant parts, were not statistically different among the replicates for rhododendron leaves (*p* = 0.36), apple fruits (*p* = 0.27), and lupine (*p* = 0.45). For each plant species, isolate ATCC 15400 exhibited a level of virulence that was significantly higher than the other 10 isolates (Figure 1). Specifically, ATCC 15400 caused lesions averaging 5.03 ± 0.15 and 27.30 ± 0.35 mm in diameter on rhododendron leaves and apple fruits, respectively, and 1.27 ± 0.04 mm in length on lupine hypocotyls. The infection of ATCC 15400 on lupines was further documented by microscopy observations. While invading the lupine hypocotyl tissue, zoospores of ATCC 15400 formed cysts and produced germ tubes 6 h after inoculation (Figures 2A,B). The germ tubes formed appressoria (Figure 2B) and expanded intercellularly and intracellularly (Figures 2C,D) 24 h after inoculation. Based on the virulence screening and microscopy observations, ATCC 15400 was determined as the most suitable isolate for the subsequent transformation. To test the sensitivity of *P. cinnamomi* ATCC 15400 to G418, *P. cinnamomi* was cultured on V8 medium with G418 concentration of 0, 10, 20, 30, 40, 50, 60, 70, 80, and 90 μg/ml, respectively. The minimum inhibitory concentration of G418 on *P. cinnamomi* growth was determined to be 70 μg/ml (Supplementary Figure 2).

### Optimize Enzyme Concentrations for Protoplast Production

**FIGURE 3:**
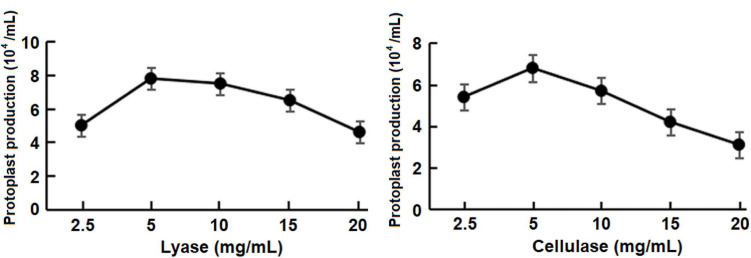
Effect of concentrations of the lysing enzyme and cellulase on the protoplast production at room temperature (approximately 25°C) with a 50-min incubation period.

For testing the concentrations of the lyase (*p* = 0.67) and the cellulase (*p* = 0.75), protoplast yield was not statistically different among three repeat test trials. Five milligrams per milliliter was determined as the optimum concentration of both enzymes that maximized the protoplast production (Figure 3) at room temperature with a 50-min incubation period.

### Transformation of *P. cinnamomi* With GFP Gene

The vector pTOR introduced into the WT included two expression cassettes: one contained the selective marker *NPTII* and the other the visual reporter *GFP*. Both genes were fused to promoter and terminator sequences of the promoter Ham34. Screening using G418 selective media, PCR detection of the *GFP* gene, and detection of fluorescence were utilized to detect successful transformations.

**FIGURE 4:**
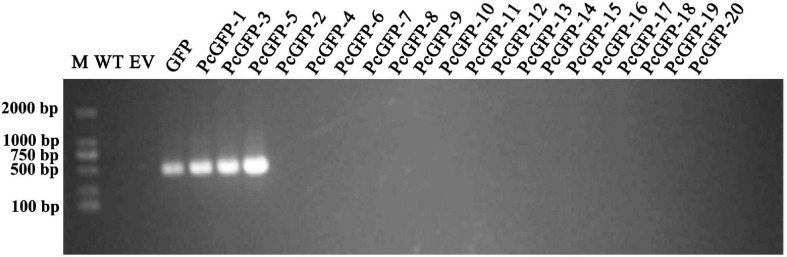
PCR assay of GFP gene integration into antibiotic-resistant transformants. M, DNA Marker DL2000; WT, genomic DNA from the wild-type isolate ATCC 15400 as a negative control; EV, the plasmid DNA from an EV plasmid as a negative control; GFP, pTOR-GFP plasmid as a positive control. Remaining lanes contain PCR products derived from genomic DNA of Pc transformants.

After three rounds of screening using G418 selective media, a total of 20 G418-resistant transformants were obtained. They were subjected for PCR verification of the presence of the *GFP* gene using genomic DNA or cDNA. As shown in Figure 4, a DNA band of about 500 nt was amplified from the positive control GFP plasmid DNA, as expected, whereas no bands were amplified from two negative controls including DNAs of the WT isolate and a mutant transformed with the EV. Three transformants exhibited the same 500-nt band, whereas the *GFP* gene was not detected in the other 17 transformants using cDNA template (Figure 4) and genomic DNA template (Supplementary Figure 3). The relative quantitative result of GFP in three transformants using qPCR is also shown in Supplementary Figure 4.

**FIGURE 5:**
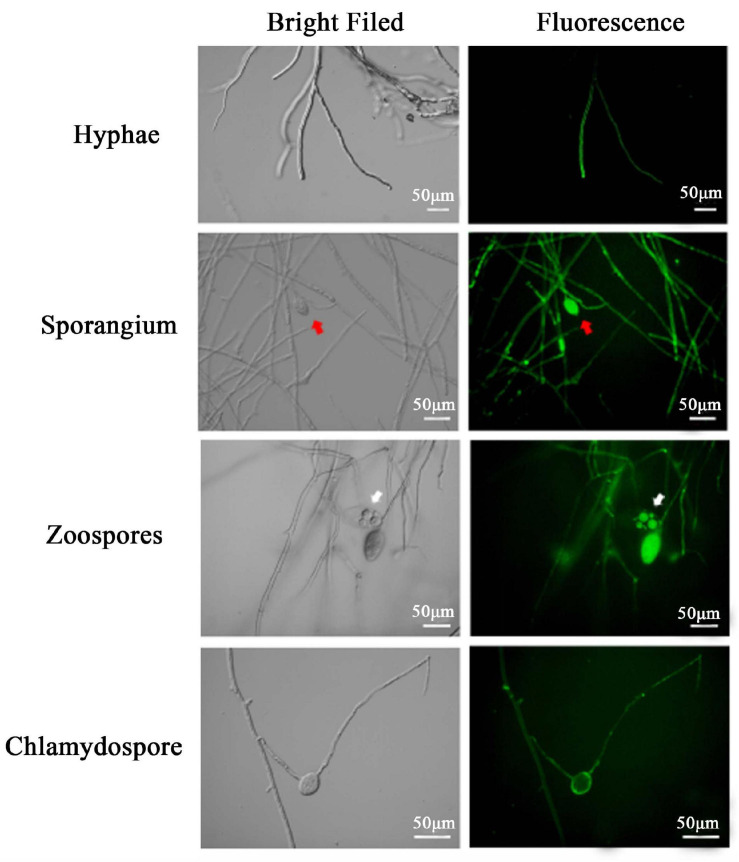
Expression of green fluorescent protein from the pTOR-GFP construct was detected as fluorescence in different asexual structures of *Phytophthora cinnamomi* transformant PcGFP-1 Red and white arrows indicate sporangia and zoosporangium, respectively.

Microscopy analyses were carried out to examine the expression of the pTOR-GFP construct in various asexual structures of the three transformants, namely, PcGFP-1, PcGFP-3, and PcGFP-5. GFP expression was observed in hyphae, sporangia, zoospores retained in cleaved sporangia, and chlamydospores of these three transformants (Figure 5 and Supplementary Figure 5). PcGFP-1 was chosen randomly for several of the following examinations on the vegetative growth and virulence.

### Vegetative Growth and Virulence of PcGFP

None of the transformants showed any abnormal morphology of the mycelia under microscopic examination. The transformant PcGFP-1 had an *in vitro* vegetative growth rate comparable to that of the WT. Radial growth rates of PcGFP-1, PcGFP-3, PcGFP-5, and WT cultured in 10% cV8A at 25°C in the dark for 3 days between two repeat experiments were not statistically different (*p* = 0.87) (Supplementary Figure 6). PcGFP-1, PcGFP-3, and PcGFP-5 had an average daily radial growth rate of 0.47 ± 0.02 mm, while that of the WT isolate was 0.45 ± 0.01 mm. We also measured sporulation rate and zoospore germination rate, which were unchanged compared to the wild type.

**FIGURE 6:**
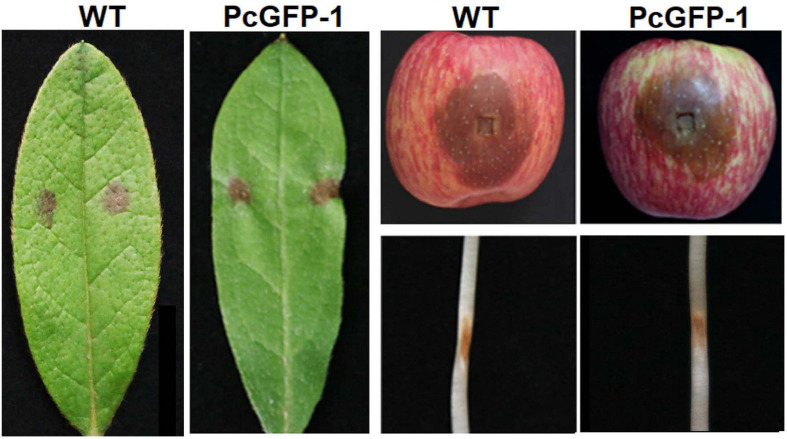
Lesions caused by *P. cinnamomi* wild-type (WT) isolate ATCC 15400 and transformant PcGFP-1 on rhododendron leaves, apple fruits, and lupine hypocotyls 3 days after inoculation.

**FIGURE 7:**
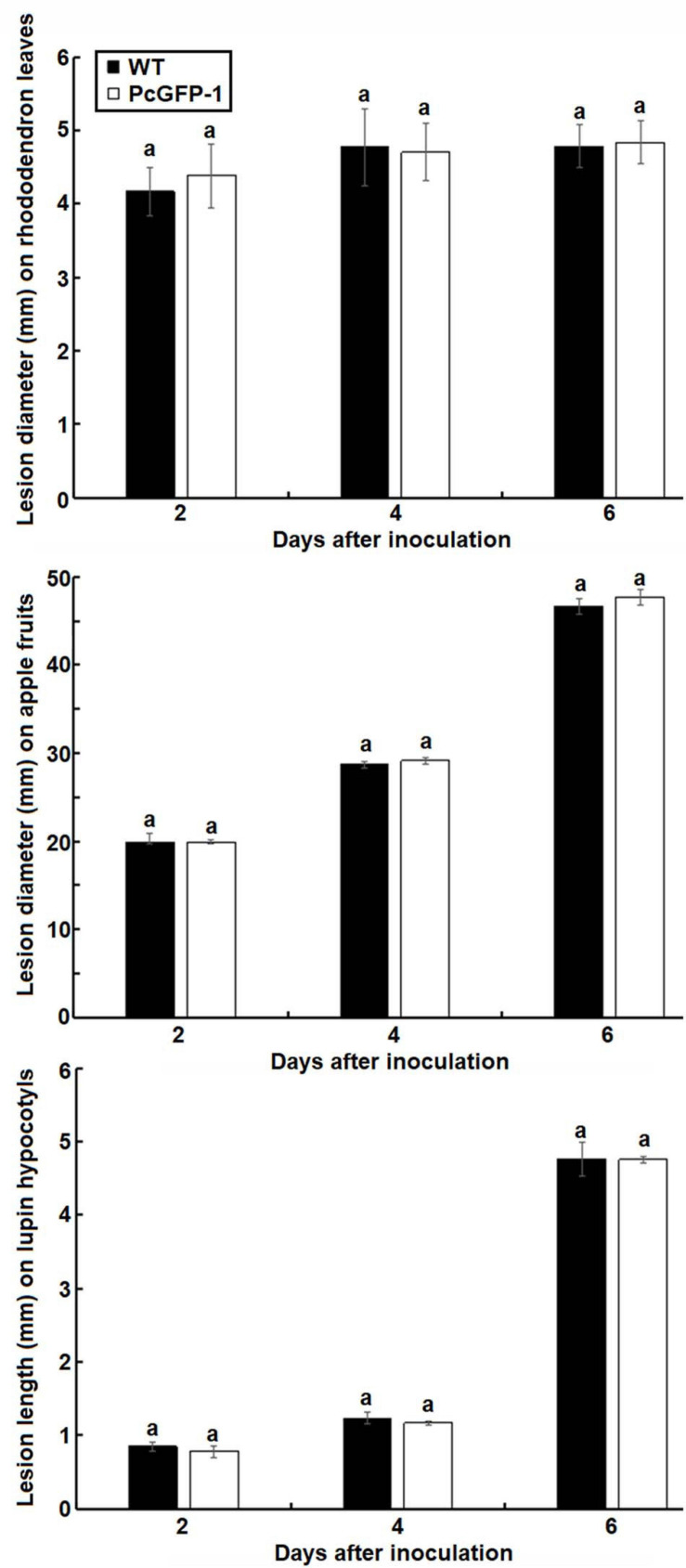
Average lesion sizes on rhododendron leaves (diameter), apple fruits (diameter), and lupine hypocotyls (length) caused by *P. cinnamomi* WT isolate ATCC 15400 and transformant PcGFP-1. The same letter above error bars indicates the absence of a significant difference between ATCC 15400 and transformant PcGFP-1 using a *t*-test.

The virulence of transformant PcGFP-1 was not compromised. Lesion sizes among three repeat experiments were the same on rhododendron leaves (*p* = 0.38), apple fruits (*p* = 0.36), and lupine hypocotyls (*p* = 0.40) (Figure 6). The average lesion sizes were not statistically different between WT and PcGFP-1 on each plant species 2, 4, or 6 days after inoculation (Figure 7).

### *In planta* GFP Expression of the Transformant PcGFP-1

**FIGURE 8:**
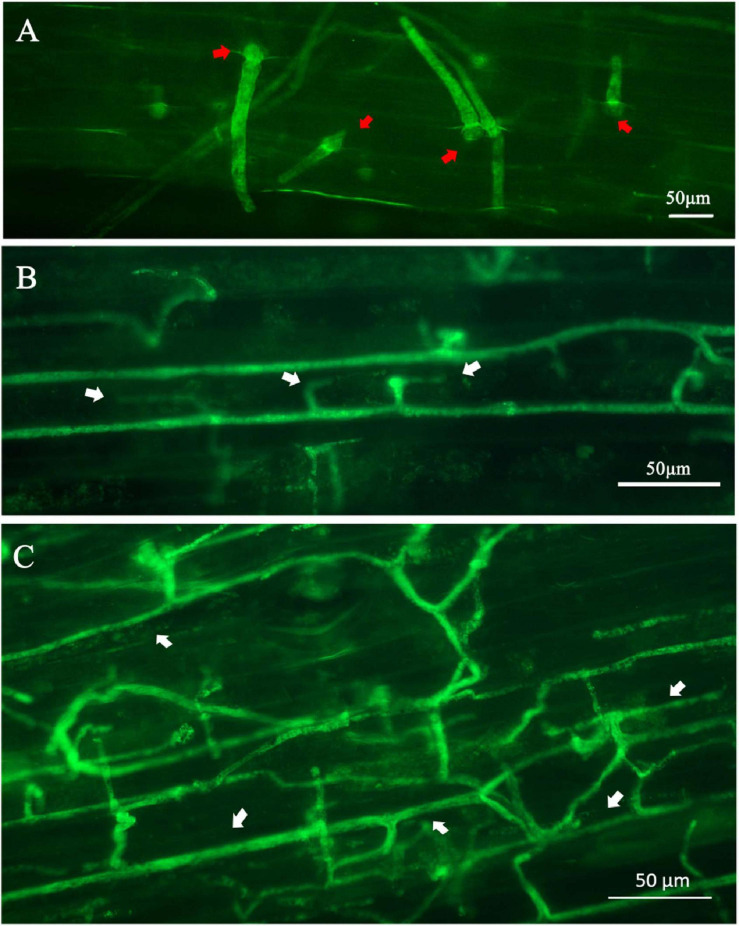
Expression of the green fluorescent protein in *Phytophthora cinnamomi* transformant PcGFP-1 during infection of lupine hypocotyls. **(A)** Fluorescence was detected in cysts (red arrows) and germ tubes 6 h after inoculation. **(B)** Hyphae with haustoria (white arrows) colonized the lupine hypocotyl cells 24 h after inoculation. **(C)** Fluorescent hyphae (white arrows) proliferating intercellularly and intracellularly 24 h after inoculation.

Fluorescent germinated cysts of the transformant PcGFP-1 were observed in inoculated lupine hypocotyl tissues 6 h after inoculation (Figure 8A). After 12 h, hyphae were observed inside lupine cells (Figure 8B) and expanded hyphae of the transformant colonized hypocotyl tissues intercellularly and intracellularly (Figure 8C).

## Discussion

An effective PEG/CaCl_2_-mediated protoplast transformation system using GFP as a reporter was established for *P. cinnamomi* in the present study. Compared to systems previously established for the same species, this improved system is able to produce a greater number of transformants without impairing the virulence of the transformants. The efficiency of the protoplast transformation system was approximately 15%. Therefore, this improved system has the potential to be used for functional analyses of *P. cinnamomi* PR genes.

Green fluorescent protein is an important reporter used for studying transcriptional regulation and subcellular localization of PR proteins, as well as morphological and subcellular structures of plant pathogenic fungi and oomycetes (Lorang et al., 2001). Because GFP can be fused to other proteins without losing its activity under *in vivo* environments, GFP fluorescent labeling is a mainstream method to study host–pathogen interactions (Lorang et al., 2001). For example, Spellig et al. (1996) labeled *Ustilago maydis* with GFP and determined that GFP was suitable for studying the interaction between this fungus and its host *Zea mays*. Kankanala et al. (2007) used GFP-fused proteins to label the rice blast fungus *Magnaporthe oryzae* and live-cell imaging to capture the development and movement of *M. oryzae* invasive hyphae within its host *Oryza sativa*. Likewise, introducing the GFP reporter gene into *Phytophthora* species has become an important method to study the infection and host interaction of these important pathogens. Thus, a genetic stable transformation system is a prerequisite for tagging *Phytophthora* using GFP. Riedel et al. (2009) randomly inserted and integrated GFP into the genome of *P. ramorum* to study the cytological morphology and the pathogenicity of GFP-tagged strains. Chen et al. (2009) used GFP as a molecular marker to observe the infection process of *P. sojae* in its host *Glycine max* and found a difference in the length of the infected germ tube of *P. sojae* between compatible and incompatible host interactions.

In this study, a GFP gene was successfully introduced and expressed in *P. cinnamomi* (Figure 4). As evidenced by microscopic examination, GFP was consistently expressed in various structures of three transformants, PcGFP-1, PcGFP-3, and PcGFP-5 (Figure 5 and Supplementary Figure 2). It is worth noting that the *GFP* gene was not detected in the other 17 isolates that continuously grew on G418-amended media during three rounds of screening (Figure 4) using cDNA template and genomic DNA template, suggesting that the gene was lost during integration of the plasmid DNA into the genome.

This study reports the first transformation of *P. cinnamomi* that successfully produced transformants with wild-type levels of vegetative growth and virulence (Figure 7). As demonstrated, the *in vitro* growth and virulence on three tested plant species of the PcGFP-1 transformant obtained in this study were not statistically different from those of the WT isolate ATCC 15400, whereas *P. cinnamomi* transformants produced in previous studies had reduced growth or virulence, or both (McCarren et al., 2005; Horta et al., 2008). Horta et al. (2008) obtained several transformants using a hygromycin selection system, but the transformants lost their ability to grow in the presence of hygromycin, except two which grew in a stable manner. It indicated that *P. cinnamomi* was difficult to transform.

The key elements of the successful system we report here were the selection of the strain and the use of G418 for selection instead of hygromycin, which, based on experience with *P. sojae*, can be toxic and permanently damage transformants in some *Phytophthora* species. We also optimized the enzyme concentration for protoplast production and the G418 concentration for selection. The improved protocol presented here was repeated three times. The efficiency of the protoplast transformation system was about 15%. There was no significant difference between transformants and wild-type strain in terms of colony morphology, diameter, and pathogenicity. The system reported here was shown to be efficient and was reproduced in two different laboratories (Nanjing Forestry University and Oregon State University). Nonetheless, its reproducibility requires further validation from different laboratories using different strains. Our improved system can be used for stable genetic transformation of *P. cinnamomi*, therefore laying the foundation for research into gene function in *P. cinnamomi*. Specifically, promoters of *P. cinnamomi* PR genes could be fused to *GFP* to examine expression of those genes during the infection process and to assess latency and tissue colonization.

## Conclusion

In conclusion, an effective PEG/CaCl_2_-mediated protoplast transformation system for *P. cinnamomi* was established in this study. By obtaining genetically stable GFP-labeled strains that retain virulence, this transformation platform lays a foundation for future studies on functional analyses of *P. cinnamomi* PR genes as well as cell biological processes of the interaction between *P. cinnamomi* and its hosts.

## Data Availability Statement

The datasets presented in this study can be found in online repositories. The names of the repository/repositories and accession number(s) can be found in the article/Supplementary Material.

## Author Contributions

TD and BT provided the experimental facilities. YX, XY, TD, FA, and BT designed the experiments. YX, BJ, and JX performed the experiments. YX analyzed the experimental data and drafted the manuscript. XY, MQ, YX, and BT revised the manuscript. All authors contributed to the article and approved the submitted version.

## Conflict of Interest

The authors declare that the research was conducted in the absence of any commercial or financial relationships that could be construed as a potential conflict of interest. The reviewer DS declared a shared affiliation, with no collaboration, with one of the authors MQ to the handling editor at the time of the review.

## Supplementary Material

Supplementary Figure 1 The oomycete expression vector pTOR was used for the PEG/CaCl2-mediated transformation of *P. cinnamomi*.

Supplementary Figure 2 *P. cinnamomi* ATCC 15400 was inoculated on V8 medium with different concentrations of G418 after 3 days growth at 25°C.

Supplementary Figure 3 PCR verification of gene integration among antibiotic-resistant transformants using genomic DNA template. M, DNA Marker DL2000; WT, the wild-type isolate ATCC 15400 as a negative control; EV, empty vector as a negative control; GFP, pTOR-GFP plasmid as a positive control.

Supplementary Figure 4 The relative quantitative result of GFP in some transformants using qPCR.

Supplementary Figure 5 Microscopic analyses of *P. cinnamomi* transformants expressing pTOR-GFP in the wild-type recipient stain (marked as PcGFP-3 or PcGFP-5, respectively). The upper panel displays the fluorescence observation from hyphae stages, the bottom panel displays the fluorescence observation from sporangia stages.

Supplementary Figure 6 Growth rates and virulence assays of PcGFP transformants. **(A,B)** Radial growth of PcGFP-1, PcGFP-3, PcGFP-5, and WT cultured in 10% cV8A after 3 days growth at 25°C. **(A)** Colony morphology. **(B)** Colony sizes averaged from 3 replicates. **(C)** Virulence assays of PcGFP-1, PcGFP-3, and PcGFP-5 on rhododendron leaves.
