# Supplementary figures and images for: An Improved Transformation System for Phytophthora cinnamomi Using Green Fluorescent Protein
Source: Front Microbiol. 2021 Jul 5;12:682754. doi: 10.3389/fmicb.2021.682754 (PMC8287854; doi:10.3389/fmicb.2021.682754)

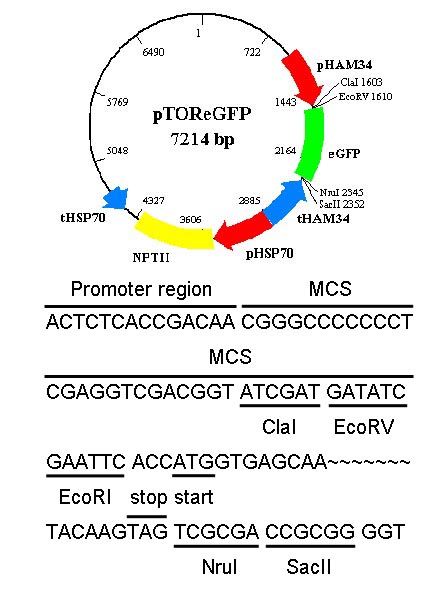

Supplement: Supplementary Figure 1 — The oomycete expression vector pTOR was used for the PEG/CaCl2-mediated transformation of P. cinnamomi. [file Image_1.JPEG]

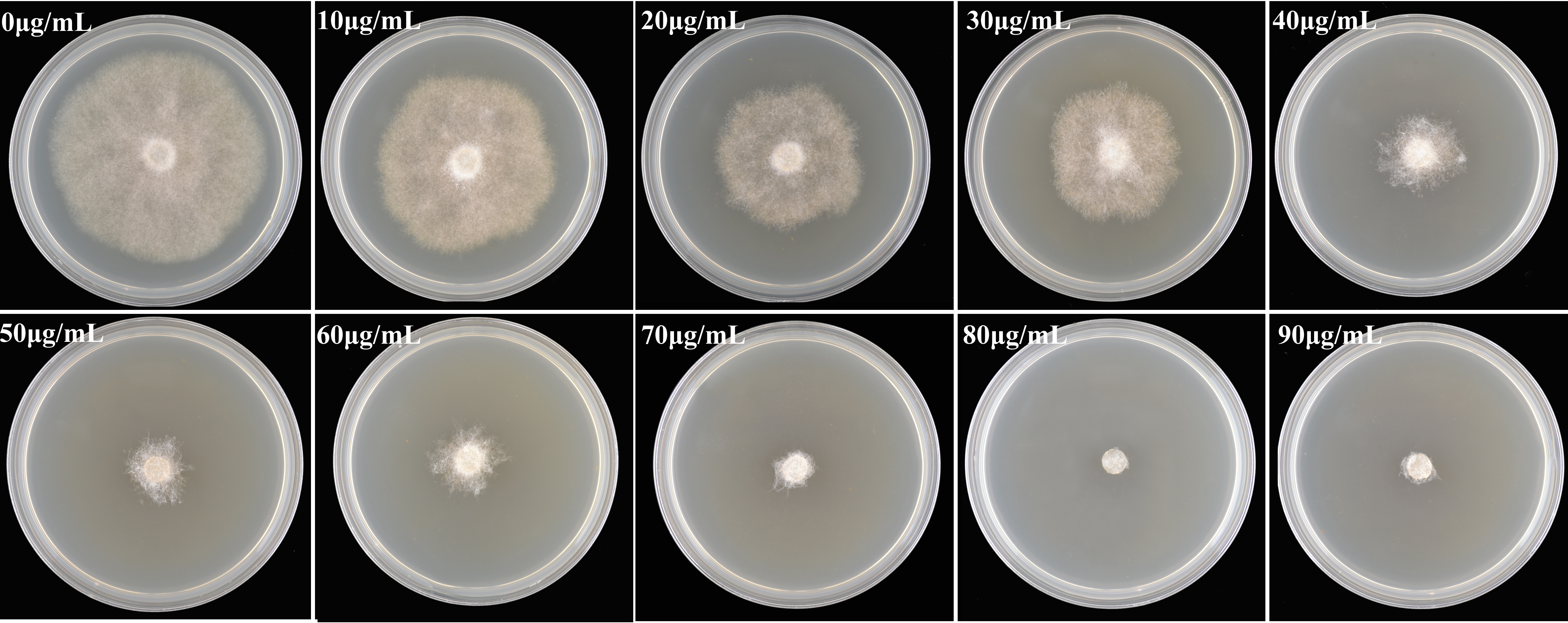

Supplement: Supplementary Figure 2 — P. cinnamomi ATCC 15400 was inoculated on V8 medium with different concentrations of G418 after 3 days growth at 25°C. [file Image_2.TIF]

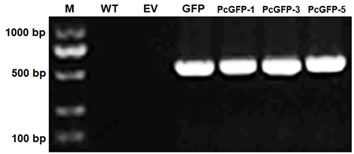

Supplement: Supplementary Figure 3 — PCR verification of gene integration among antibiotic-resistant transformants using genomic DNA template. M, DNA Marker DL2000; WT, the wild-type isolate ATCC 15400 as a negative control; EV, empty vector as a negative control; GFP, pTOR-GFP plasmid as a positive control. [file Image_3.TIF]

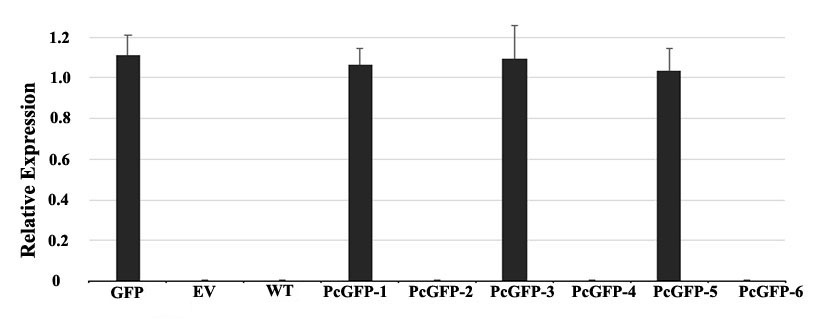

Supplement: Supplementary Figure 4 — The relative quantitative result of GFP in some transformants using qPCR. [file Image_4.JPEG]

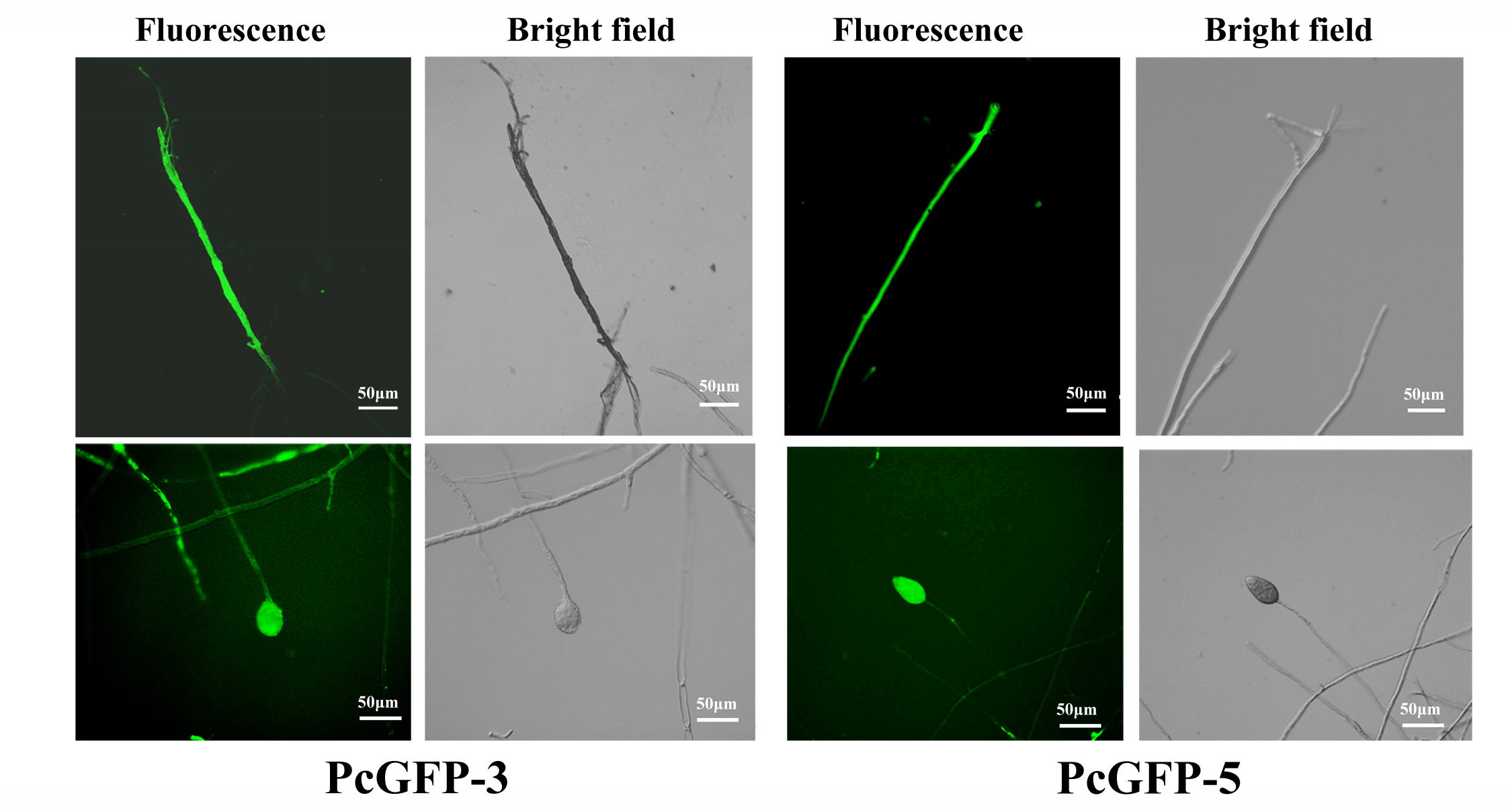

Supplement: Supplementary Figure 5 — Microscopic analyses of P. cinnamomi transformants expressing pTOR-GFP in the wild-type recipient stain (marked as PcGFP-3 or PcGFP-5, respectively). The upper panel displays the fluorescence observation from hyphae stages, the bottom panel displays the fluorescence observation from sporangia stages. [file Image_5.TIF]

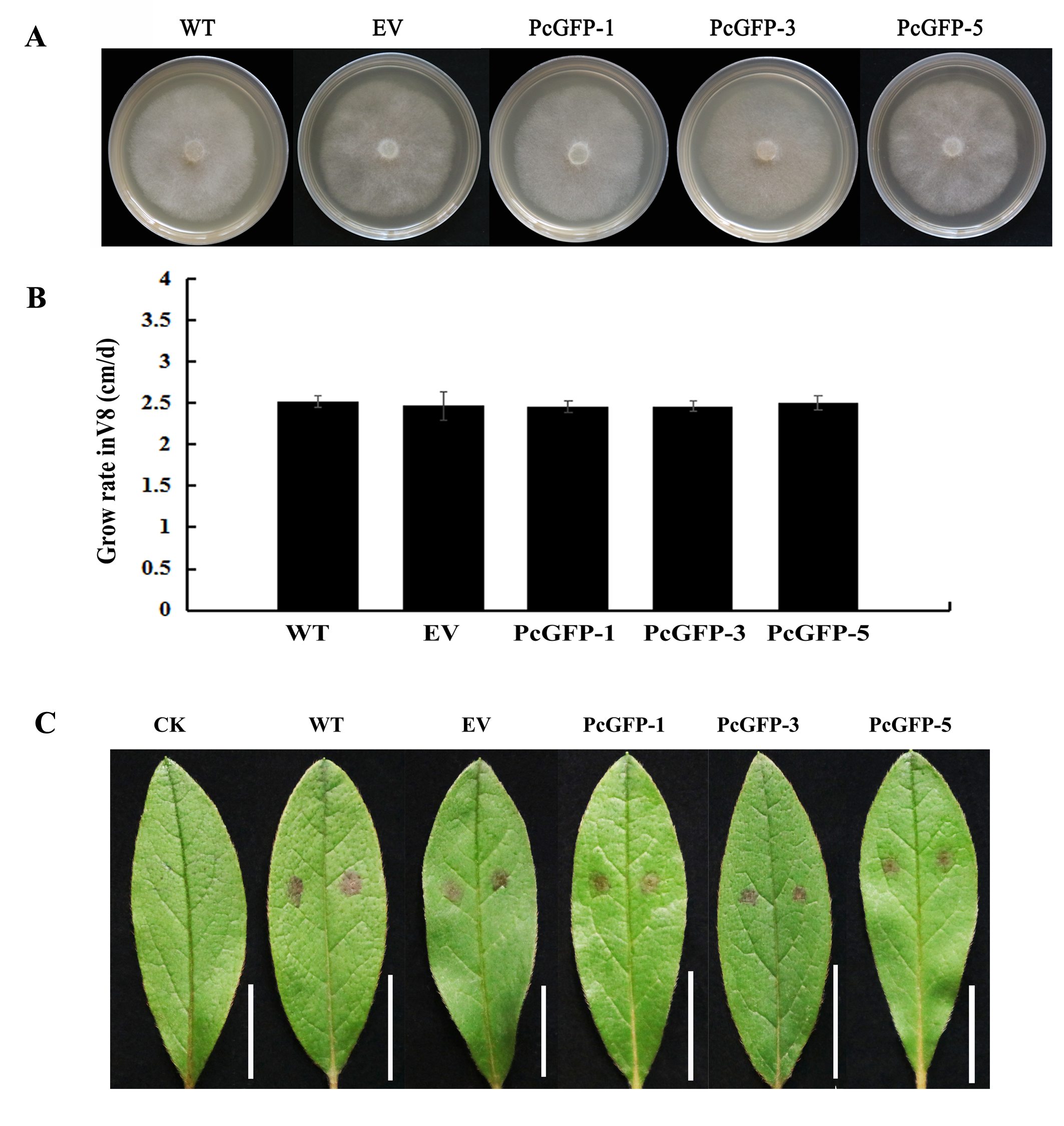

Supplement: Supplementary Figure 6 — Growth rates and virulence assays of PcGFP transformants. (A,B) Radial growth of PcGFP-1, PcGFP-3, PcGFP-5, and WT cultured in 10% cV8A after 3 days growth at 25°C. (A) Colony morphology. (B) Colony sizes averaged from 3 replicates. (C) Virulence assays of PcGFP-1, PcGFP-3, and PcGFP-5 on rhododendron leaves. [file Image_6.TIF]
